# Short IQCODE as a screening tool for MCI and dementia: Preliminary results

**DOI:** 10.1590/S1980-57642009DN20400012

**Published:** 2008

**Authors:** Tíbor Rilho Perroco, Antonio Eduardo Damin, Norberto A. Frota, Mari-Nilva M. Silva, Viviane Rossi, Ricardo Nitrini, Cássio M.C. Bottino

**Affiliations:** CEREDIC and Old Age Research Group (PROTER), Department of Neurology and Institute and Department of Psychiatry, Faculty of Medicine, University of São Paulo.

**Keywords:** dementia, diagnosis, cognitive test, functional scales, screening tests, informant report

## Abstract

Reaching a diagnosis may be difficult in the initial stages of dementia, especially in low educated individuals, when informant reports may be useful.

**Objectives:**

To compare the sensitivity and specificity of the IQCODE against cognitive tests applied in clinical practice and to evaluate the possible cut-off points in Brazil.

**Methods:**

Individuals without dementia (CDR=0; N=5), with Mild Cognitive Impairment (MCI) (CDR=0.5; N=15) and demented (CDR≥1; N=29) were evaluated using the Short IQCODE, a 16-item questionnaire applied to an informant, and on standard cognitive and functional scales. Diagnosis was reached by a consensus team with expertise in dementia, according to DSM-IV criteria, which was blind to the IQCODE results.

**Results:**

IQCODE scores were positively correlated to the CDR (r=0.65, p<0.001) and negatively correlated with years of schooling (r= –0.33, p=0.021). IQCODE scores were positively correlated with CDR controlled by age and education (r=0.61, p<0.001). Linear regression showed that age was associated with the IQCODE (p=0.016) whereas education was not associated (p=0.078). IQCODE means according to the CDR classification were: CDR 0–3.37; CDR 0.5–3.75; CDR 1–4.32; CDR 2–4.61; CDR 3–5.00. The area under the ROC curve for dementia vs. controls was 0.869 (p<0.001), MCI vs. controls, 0.821 (p<0.001); and according to the groups classified by the CDR was: CDR 0.5 vs. CDR 1=0.649 (p=0.089), CDR 1 vs. CDR 2=0.779 (p=0.009), and CDR 2 vs. CDR 3=0.979 (p=0.023).

**Conclusions:**

These preliminary findings suggest that the short IQCODE can be used for the screening of MCI and dementia in Brazil.

## Abstract (pt)

O diagnóstico pode ser difícil em estágios iniciais de demência, especialmente em população de baixa escolaridade, quando os relatos de informantes podem ser úteis.

**Objetivo:**

Comparar a sensibilidade e especificidade do IQCODE com outros testes e diagnóstico clinico, assim como identificar os possíveis pontos de corte no Brasil.

**Métodos:**

Indivíduos sem demência (CDR=0; N=5), com comprometimento cognitivo leve (MCI) (CDR=0,5; N=15) e dementados (CDR≥1; N=29) foram avaliados usando o IQCODE reduzido, um questionário de 16 itens aplicado ao informante, e escalas cognitivas e funcionais padrões. Diagnóstico final foi fornecido por consenso de especialistas, com experiência em demência, de acordo com os critérios do DSM-IV, e cegos quanto ao resultado do IQCODE.

**Resultados:**

IQCODE tem uma correlação positiva com o CDR (r=0.65, p<0.001) e negativa com anos de escolaridade (r= –0.33, p=0.021). IQCODE mantêm correlação positiva com o CDR quando controlado por idade e escolaridade (r=0.61, p<0.001). Na regressão linear a idade continuou a afetar o resultado do IQCODE (p=0.016) enquanto a escolaridade não (p=0.078). Médias do IQCODE de acordo com a classificação do CDR: CDR 0–3.37; CDR 0.5–3.75; CDR 1–4.32; CDR 2–4.61; CDR 3–5.00. A área sob a curva ROC para demência vs. controles foi 0.869 (p<0.001), MCI vs. controles, 0.821 (p<0.001) e de acordo com os CDR foi: CDR 0.5 vs. CDR 1=0.649 (p=0.089), CDR 1 vs. CDR 2=0.779 (p=0.009) e CDR 2 vs. CDR 3 =0.979 (p=0.023).

**Conclusão:**

Esses achados preliminares sugerem que o IQCODE Curto pode ser usado como instrumento de triagem para MCI e demência no Brasil.

Screening tests are essential in the investigation of patients with suspected dementia. As the population ages and dementia prevalence and incidence rates increase,^1^ such tests have become even more important.

Some screening tests entail cognitive assessments performed directly with the patient while others are based on information provided by the proxy (or family member).^2^ Studies have shown that the association of both cognitive test and informant report is an effective dementia screening method,^3,4^ yielding increased sensitivity and specificity of the instruments than when used independently. However, in some cases the patient is unable to provide information properly, due to severe disease, refusal to cooperate, or because the patient has a very low educational level.^5,6^ In such cases, indirect assessments, performed only with the informant, play a major role in clinical practice, especially in developing countries where the mean educational level is often lower than that found in developed countries.^6^

Ideally, screening tests must be brief, easily adapted, with a high reproducibility level between different raters and cultures, and show little influence from educational level. Unfortunately, many of the current tests do not meet these premises, or are long and often require training prior to their application.^7^ In this context, a brief screening test applied to the informant, the IQCODE,^8^ was evaluated to verify the efficacy of its long and short versions adapted for use in Brazil.

The IQCODE was originally developed as an interview with informant.^9^ A total of 39 initial items were reduced to 26, making the test faster and more suited for application. The informant is asked about issues regarding changes in the subject’s memory, cognition and language ability over the last ten years. The items on the IQCODE are rated on a five-point scale, where 1 indicates “much better” and 5 indicates “much worse”. The final score is the average of the rated item scores.

There are versions in numerous languages, including Chinese, German, Finnish, French, Canadian French, Dutch, Italian,^10^ Japanese, Korean, Norwegian, Polish, Spanish, and Thai.^11^ Many short versions have been evaluated, with differences in number of items. Sixteen-item versions in English^12^ and German^13^ and a 17-item version in Spanish^14^ have been validated.

This aim of this preliminary report was to evaluate the performance of a 16-item, short (S) Brazilian version of the Informant Questionnaire on Cognitive Decline in the Elderly (IQCODE), to compare against cognitive tests and functional scales, and to investigate the possible cut-off points that can be applied in clinical practice for the Brazilian population.

## Methods

A cross-sectional, case-control study was performed in patients diagnosed with dementia, MCI^15^ along with controls, at the CEREDIC, Clinicas Hospital, School of Medicine, University of São Paulo.

Informants of individuals without dementia (CDR=0; N=5), with Mild Cognitive Impairment (MCI) (CDR=0.5; N=15) and patients with dementia (CDR=1; N=29) were evaluated using the Short version of the IQCODE (IQCODE-S), which consists of a reduced 16-item version of the original questionnaire. Besides the IQCODE-S, all participants were evaluated with the Brazilian versions of the Mini-Mental State Examination (MMSE),^16^ cognitive test of the CAMDEX interview (CAMCOG),^17^ Clinical Dementia Rating (CDR), Brief Cognitive Battery, while their informants were evaluated with the Pfeffer-Functional Activities Questionnaire and the Neuropsychiatric Inventory (NPI).^18^ Participants with suspected dementia were submitted to a work up for dementia (blood tests and neuroimaging scan) according to the recommendations suggested by the Brazilian Academy of Neurology.^19^ Final consensus diagnosis was performed by at least one neurologist, one psychiatrist and one geriatrician with expertise in dementia, according to DSM-IV (APA, 1994)^20^ and NINCDS/ADRDA criteria. The Petersen criteria (1999)^15^ was applied to classify subjects with MCI in our sample who were all classified as CDR=0.5. The consensus team was blind to the IQCODE results obtained through the informants.

The Hospital Ethics Committee approved this study and all the patients and informants agreed to participate in the study after signing an informed consent term.

Data analysis was performed using the statistical package SPSS for Windows version 14.0. We presented descriptive analysis, analysis of receiver operating characteristic (ROC) curves and logistic regression.^21,22^ Diagnosis of dementia was the dependent variable, and the MMSE and IQCODE versions were the independent variables for the regression analysis.

## Results

The sample evaluated comprised 49 subjects (male=25; female=24), 9 of whom were classified as CDR=0, 11 as CDR=0.5, and 29 as CDR≥1, with mean age=70.5 years, and mean schooling=5.7 years.

Means on the IQCODE-S by CDR groups are shown in Table 1.

**Table 1:** Mean scores on the short version of the Brazilian IQCODE according to CDR classification.

n	CDR	IQCODEMean (SD)
10	0	3.37 (0.90)
11	0.5	3.75 (0.42)
17	1	4.32 (0.39)
9	2	4.61 (0.33)
2	3	5.00 (-)

Our results showed that IQCODE-S scores were positively correlated to CDR (r=0.65, p<0.001) and negatively correlated with years of schooling (r= –0.33, p=0.021). Also, IQCODE scores were positively correlated with CDR controlled by age and education (r=0.61, p<0.001). Linear regression showed that age was associated with the IQCODE scores (β=0.26, p=0.016) while education was not (β= –0.19, p=0.078). The area under the ROC curve comparing demented subjects to the control group was 0.869 (p<0.001), comparing MCI to controls was 0.821 (p<0.001) and according to the groups classified by the CDR was: CDR 0.5 vs. CDR 1=0.649 (p=0.089), CDR 1 vs. CDR 2=0.779 (p=0.009), and CDR 2 vs. CDR 3, p=0.023.

**Graph 1:**
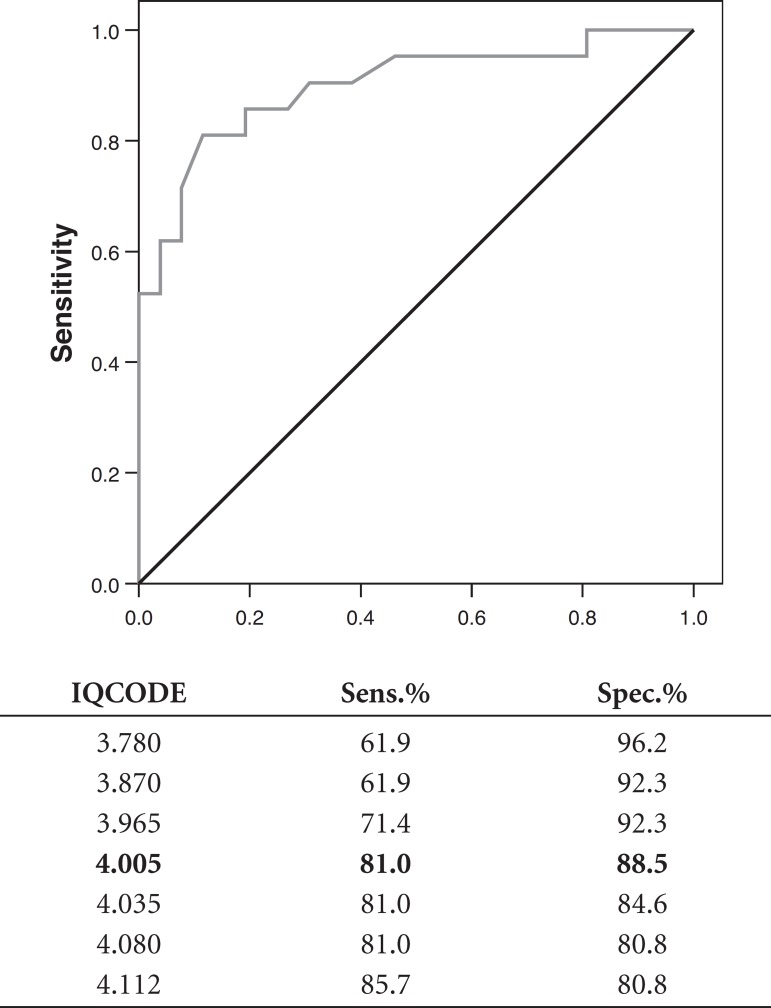
ROC Curve comparing CDR=0 and 0.5, to CDR≥1.

**Graph 2:**
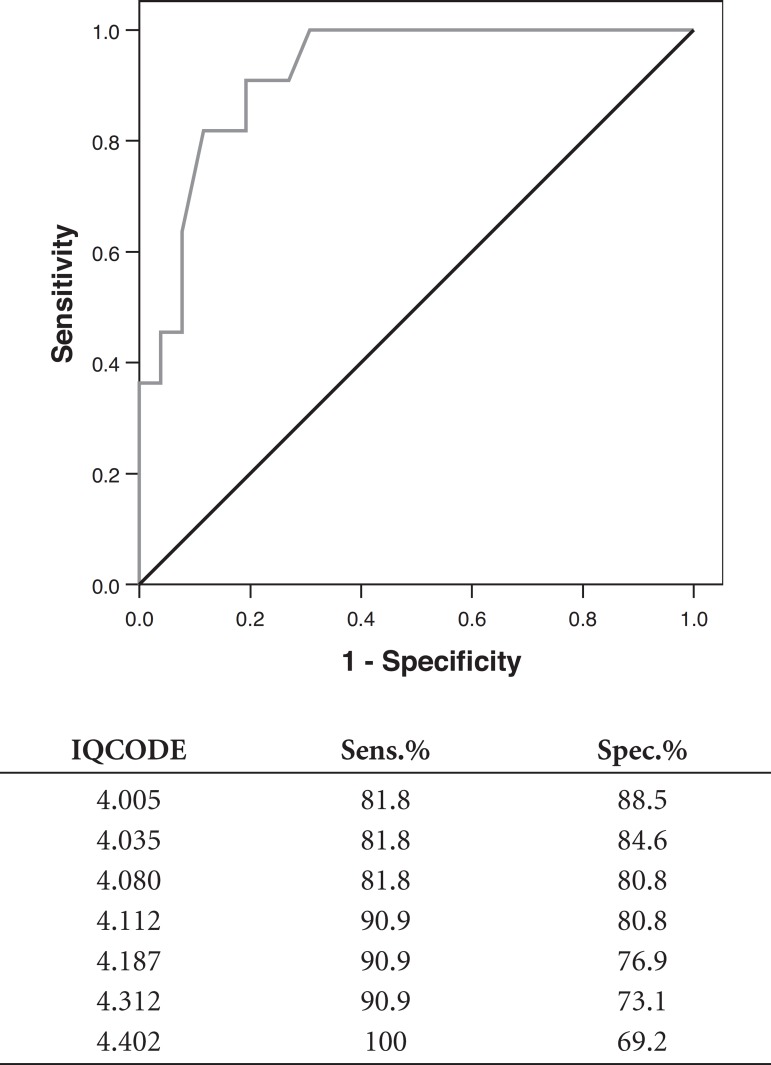
ROC Curve comparing CDR=0.5 to CDR≥1.

The IQCODE-S area under the ROC curve, including subjects CDR=0 and 0.5 compared to those with CDR≥1 was 0.898 (p≤0.001). Comparing those subjects classified as CDR=0.5 to those considered CDR≥1, the area under the curve was 0.921 (p≤0.001) as presented in Graphs 1 and 2 below.

## Discussion

In the present report, the IQCODE-S applied to informants of low educated elderly subjects showed high sensitivity and specificity values in differentiating subjects with MCI and mild to moderate dementia from normal controls. Educational level was found not to influence the IQCODE-S version.

Using the Spanish version of the short IQCODE, Morales et al. (1997) compared the instrument in two different samples, one urban and the other rural.^23^ The authors did not report the educational level of the rural sample, but 78.2% of these individuals had no formal education. Mean educational level was 4.9 years for the urban sample. The IQCODE was better than the MMSE in both samples and unlike the MMSE, demonstrated no influence of age, educational or cultural level.^23^

The IQCODE-S cut-off points found in the present study ranged from 4.0 to 4.1, which are slightly higher than those suggested in the literature,^12,24-26^ as can be seen in Table 3. However, we included patients with MCI and very mild dementia in our sample which probably explains these results.

**Table 3:** Results using short versions of the IQCODE.

Author (year of publication)	Cutoff scores	Sensitivity	Specificity	ROC Curveareas
Jorm (1994)^24^	≥3.38	79%	82%	0.85
Jorm et al. (1996)^12^	≥3.38	75%	68%	0.77
Del-Ser et al. (1997)^25^	≥3.88	79%	73%	0.77
Harwood et al. (1997)^26^	≥3.44	100%	86%	NA

NA, Not available.

Isella et al. (2006) followed up subjects with MCI for at least 2 years or until they converted to dementia.^27^ The ability to differentiate patients with MCI from healthy controls proved similar for the IQCODE (area under the curve – AUC=0.86) and the MMSE (AUC=0.84). These areas were also very similar to the ones found in the present study (AUC=0.821, p=0.001). Isella et al. evaluated the independent predictive ability of IQCODE and memory scores using logistic regression, demonstrating that the IQCODE alone achieved the best correct classification of 81%.^27^

In Brazil, Areza-Fegyveres et al. (2008) described the applicability of the IQCODE in a population with low schooling, reporting that the proxy-reports were similar to the elderly reports.^28^ This finding lends support to the efficacy of the test and suggests another possible use for the IQCODE.

Bustamante et al. (2003) showed that the combination of a cognitive test with a functional scale can improve the detection of mild to moderate cases of dementia, even in samples of very heterogeneous populations with regard to socioeconomic status and educational level.^29^ These results suggested that a functional scale combined with a cognitive test can improve the diagnostic accuracy of dementia.

Limitations of the study include a relatively small sample, with low mean educational level, although containing few elderly individuals with no formal education. Data on the educational level of relatives or proxies were not collected, and this point should be addressed in further studies since it may have influenced the IQCODE results.

In summary, the results suggest that the short Brazilian version of the IQCODE can be used for the screening of MCI and mild cases of dementia. The IQCODE is not influenced by educational level, which makes it an adequate instrument for use in our country and useful for other samples with a similar educational profile.

## Figures and Tables

**Table 2:** Correlation between the IQCODE, sociodemographic and clinical variables.

			Schooling	Age
Spearman's rho	Schooling	Correlation coefficient	1.000	-0.045
		Sig. (2-tailed)	-	0.760
		N	48	48
	Age	Correlation coefficient	-0.045	1.000
		Sig. (2-tailed)	.760	-
		N	48	48
	CDR	Correlation coefficient	-0.219	0.355*
		Sig. (2-tailed)	0.143	0.015
		N	46	46
	IQCODE	Correlation coefficient	-0.209	0.429**
		Sig. (2-tailed)	0.153	0.002
		N	48	48

* Correlation is significant at the 0.05 level (2-tailed);

** Correlation is significant at the 0.01 level (2-tailed); CDR, Clinical Dementia Rating; IQCODE, Informant Questionnaire on Cognitive Decline in the Elderly
